# Role of Key Residues at the Flavin Mononucleotide (FMN):Adenylyltransferase Catalytic Site of the Bifunctional Riboflavin Kinase/Flavin Adenine Dinucleotide (FAD) Synthetase from *Corynebacterium ammoniagenes*

**DOI:** 10.3390/ijms131114492

**Published:** 2012-11-08

**Authors:** Ana Serrano, Susana Frago, Adrián Velázquez-Campoy, Milagros Medina

**Affiliations:** 1 Department of Biochemistry and Cellular and Molecular Biology, Faculty of Sciences, Institute for Biocomputation and Physics of Complex Systems (BIFI)-Joint Unit BIFI-IQFR (CSIC), University of Zaragoza, Pedro Cerbuna, 12. 50009 Zaragoza, Spain; E-Mails: anaserra@unizar.es (A.S.); susana.frago@path.ox.ac.uk (S.F.); adrianvc@unizar.es (A.V-C.); 2 ARAID Foundation, Aragonian Government, 50018 Zaragoza, Spain

**Keywords:** FAD synthetase, ATP:riboflavin kinase, ATP:FMN adenylyltransferase, FAD pyrophosphorylase, site-directed mutagenesis, catalytic activity, substrate binding

## Abstract

In mammals and in yeast the conversion of Riboflavin (RF) into flavin mononucleotide (FMN) and flavin adenine dinucleotide (FAD) is catalysed by the sequential action of two enzymes: an ATP:riboflavin kinase (RFK) and an ATP:FMN adenylyltransferase (FMNAT). However, most prokaryotes depend on a single bifunctional enzyme, FAD synthetase (FADS), which folds into two modules: the *C*-terminal associated with RFK activity and the *N*-terminal associated with FMNAT activity. Sequence and structural analysis suggest that the 28-HxGH-31, 123-Gx(D/N)-125 and 161-xxSSTxxR-168 motifs from FADS must be involved in ATP stabilisation for the adenylylation of FMN, as well as in FAD stabilisation for FAD phyrophosphorolysis. Mutants were produced at these motifs in the *Corynebacterium ammoniagenes* FADS (*Ca*FADS). Their effects on the kinetic parameters of *Ca*FADS activities (RFK, FMNAT and FAD pyrophosphorilase), and on substrates and product binding properties indicate that H28, H31, N125 and S164 contribute to the geometry of the catalytically competent complexes at the FMNAT-module of *Ca*FADS.

## 1. Introduction

Flavin mononucleotide (FMN) and flavin adenine dinucleotide (FAD) are essential cofactors for numerous enzymes (*i.e.*, dehydrogenases, monooxygenases, oxidases, oxido-reductases) contributing to one- and two-electron oxido-reduction processes critical to the major metabolic energy transformation routes [1–3]. Riboflavin (RF), the precursor of FMN and FAD, can be *de novo* synthesised by plants, fungi and many bacteria, but in mammals the only known RF source is the diet (vitamin B_2_) [4–6]. Conversion of RF into FMN and FAD is catalysed by the sequential action of two enzymatic activities: an ATP:riboflavin kinase (RFK, EC 2.7.1.26) transforms RF and ATP into FMN, and an ATP:FMN adenylyltransferase (FMNAT, EC 2.7.7.2) catalyses the adenylylation of FMN into FAD using a second molecule of ATP (Figure S1a). In mammals, yeast and some archaea, these two activities reside in two different proteins [7–14], but in most prokaryotes they are encoded by a gene that produces a single bifunctional protein, FAD synthetase (FADS) [9,15,16].

FADS from *Corynebacterium ammoniagenes* (*Ca*FADS) folds into two modules, each one related to one of the activities [17,18] (Figure S1b). The *N*-terminal module (residues 1–186, FMNAT-module) forms an α/β dinucleotide binding domain with a typical Rossmann fold, consisting of a twisted parallel β-sheet of six strands and five α-helices distributed at both sides of the β-sheet, and ends in a small subdomain built by a β-hairpin and two short α-helices. It does not present sequence or structural homology with yeast and mammal FMNATs, but belongs to the nucleotidyltransferase (NT) superfamily [18]. Thus, the *N*-terminal module was assigned with the FMNAT activity. In some species, such as *C. ammoniagenes*, this module also catalyses a FAD pyrophosphorylase (FADpp) activity, producing FMN and ATP from FAD and pyrophosphate (PPi). The *C*-terminal module (residues 187–338, RFK-module) shares sequence and structural homology with monofunctional RFKs, and catalyses this activity [19,20]. It folds in a globular domain consisting of a β-barrel with six antiparallel β-strands, a long α-helix and seven loops connecting them. Despite the identification of independent binding sites for adenine and flavin nucleotides in each module [18,20,21], the FMNAT-module does not appear to be self-sufficient in transforming FMN into FAD [17]. The *Ca*FADS crystal structure is also organised in a hexameric arrangement formed by the interaction of two trimers, *i.e*., a dimer of trimers. In each trimer the protomers are connected in a head-to-tail disposition that might allow ligand channelling between the RFK and FMNAT active sites of contiguous protomers [9,18].

**Figure 1:**
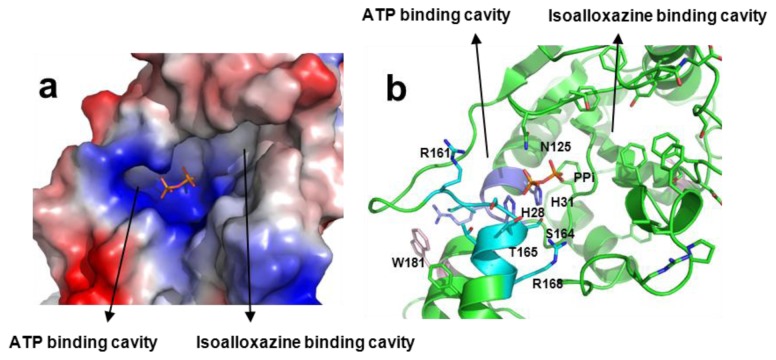
Active site at the ATP:FMN adenylyltransferase (FMNAT)-module of *Ca*FADS (PDB code 2X0K), showing (**a**) details of the surface electrostatic potential and (**b**) position of relevant side chains (shown in sticks). Residues from the consensus 28-HxGH-31, 123-Gx(D/N)-125 and 161-xxSSTxxR-168 motifs are shown with carbons in violet, green and cyan, respectively. Both panels show the position of the PPi product (phosphates in orange) as observed in the crystal structure.

The FMNAT-module contains three motifs highly conserved in the FADS and NT families; namely, 28-HxGH-31, 123-Gx(D/N)-125 and 161-xxSSTxxR-168 [9,16,17] (Figure S1c). Histidine residues of the HxGH motif have been analysed in several monofunctional NTs [22–26], suggesting they might stabilise the phosphates and adenine moiety of ATP during catalysis [18,25–31]. Nevertheless, conclusions are not clear on the particular role of each His and vary in each particular species [23,26]. There are no previous site-directed mutagenesis reports for the role of residues from the second motif, 123-Gx(D/N)-125, but its similarity with the motif found at the active site of class 1 aminoacyl-tRNA synthetases [13], and its location in the putative active site in *Ca*FADS, suggests it might have an important role in the adenylylation reaction (Figure 1) [18]. S164 and T165, highly conserved in the 161-xxSSTxxR-168 consensus motif, have been proposed to stabilise the adenine, and β-P and γ-P of ATP [18,22,30]. Finally, R161, although not conserved in the sequences of FADSs or NTs, might stabilise the adenine and phosphate groups [17,18,28].

The *Ca*FADS structure was reported to be in a complex with the PPi product of the FMNAT reaction. PPi is situated in a large open cavity where substrates and products for the FMNAT and FADpp reactions have been proposed to bind (Figure 1a) [18]. The side chains of H28, H31, H57, N125, R161 and R168 contribute to the cavity’s positively charged surface, with the position of PPi stabilised via direct contact with the side chains of H28, H31, N125 and S164 (Figure 1b). Therefore, the structural data suggest a catalytic role for these residues. No functional data are available regarding their role in bifunctional FADSs, but a preliminary qualitative mutational analysis in *Ca*FADS suggested some of them might be crucial for FMNAT activity [17]. To evaluate the effects of the interactions provided by these polar and positively charged residues on the kinetic parameters of *Ca*FADS, we have analysed here the properties of mutants where polarity and charge have either been removed (Ala mutants) or inverted (Asp mutants). Mutagenesis of these motifs permitted a better understanding of the FMNAT and FADpp catalytic mechanism.

## 2. Results and Discussion

### 2.1. Expression, Purification, and Spectral Properties of the CaFADS Mutants

The expression level for most of the mutants was similar to that of wild-type (WT) *Ca*FADS (2–5 mg protein/g cells). However, H28A *Ca*FADS showed surprisingly low expression (<1 mg protein/g cells), while more than 10 mg protein/g cells were obtained for the S164 variants. In contrast to the WT protein samples, which, contain small amounts of oligomeric species easily separated by gel filtration chromatography [18,20], the H31D variant failed to show a monomeric discrete form.

All the mutants retained the main secondary structural elements of WT *Ca*FADS (Figure S2a). Their near-UV circular dichroism (CD) spectra showed a broad negative band between 260 and 300 nm with two discrete minima at 284 and 291 nm, as for WT *Ca*FADS [21] (Figure S2b), but replacements of H31, N125 and R161 by Ala and of H31 and N125 by Asp decreased the intensity of the signal (Figure S2b). The two tryptophan residues at the FMNAT-module of *Ca*FADS are partially accessible to the solvent. In particular, W181 is relatively close to the putative adenine binding site of ATP, where mutations were introduced (Figure 1b), suggesting they might modify its solvent accessibility.

Addition of saturating concentrations of RF to *Ca*FADS or to the *Ca*FADS:ADP complex for the different variants induced difference spectra in the visible region similar to those for the WT form, indicating interaction of the flavin isoalloxazine ring with the protein (Figure S3a,b). More appreciable changes, both in shape and intensity, were observed upon addition of FMN or FAD to *Ca*FADS (Figure S3c,e), and no difference spectra at all were detected for the R161D variant. Since in the absence of adenine nucleotides both flavins are expected to bind only at the FMNAT-module [20,21], the mutations appear to modulate the isoalloxazine: protein interaction at this module. FMN addition to the preformed *Ca*FADS:ADP complexes increased the difference spectra magnitude (Figure S3f), in agreement with the reported detection of an additional FMN binding site in the RFK-module [20,21]. Mutations also modified the relative intensity of the spectral bands upon addition of FAD (only able to bind at the FMNAT-module) to the preformed *Ca*FADS:ADP complexes with respect to both, the WT *Ca*FADS:ADP complex and the difference spectra in the absence of ADP (Figure S3c,d). Since the mutated positions are, in principle, far from the expected allocation site for the flavin isoalloxazine at the FMNAT-module (see Figures 1 and 5 below), these results suggest that changes in the conformation of the adenine nucleotide binding pocket induce changes either in the isoalloxazine location within the protein or in its binding cavity.

### 2.2. Stability of the CaFADS Mutants

Thermal denaturation of WT *Ca*FADS by differential scanning calorimetry (DSC) showed two overlapping transitions (data not shown) with an apparent unfolding mid-transition temperature (*T*_m,app_) of 42.5 °C (Table S1), suggesting that the unfolding processes of the FMNAT- and RFK-modules are not fully cooperative [21]. Replacement of N125, S164 and T165 by Ala produced a 2–3 °C decrease in the *T*_m,app_, indicating a destabilising effect. However, mutations H28A, H31A, R161A, R161D, S164D and T165D stabilised the enzyme with increases of *T*_m,app_ up to 6–7 °C. H28D and N125D had similar *T*_m,app_ values to WT *Ca*FADS (Table S1). Therefore, replacement of polar residues in this cavity produces different effects, which apparently depend on the protein environment stabilising the new side chain, on its ability to provide or prevent specific salt-bridges or dipole-dipole contacts with respect to the WT enzyme, as well as on different solvation patterns in the substrate binding cavity. Thus, changes in the polarity of residues at the active site cavity of the FMNAT-module modulate its shape, interacting properties and stability. Therefore, we cannot dismiss replacements, particularly with Asp, resulting in adventitious effects unrelated to the enzyme catalytic mechanism.

### 2.3. Effects of Mutations on the FMNAT Activity of CaFADS

The values of *k*_cat_, *K*_m_^FMN^, and *K*_m_^ATP^ for the FMNAT activity of WT *Ca*FADS are 17 min^−1^, 1.2 μM, and 36 μM, respectively (Table 1), in agreement with data previously reported [9,32]. In general, mutations at H28, H31, N125, R161, S164 and T165 had a deleterious impact on activity.

**Figure 2:**
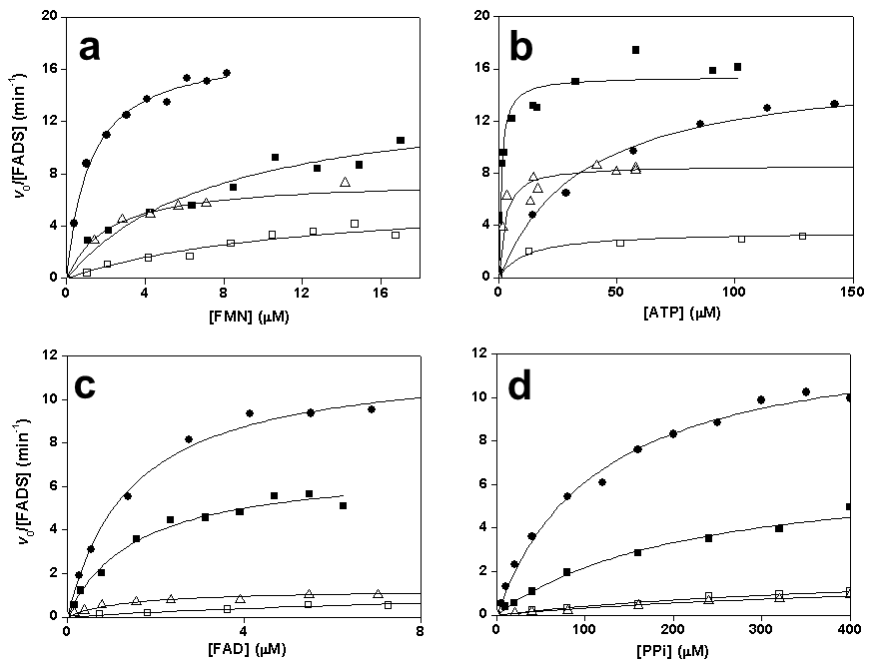
Steady-state rates for the FMNAT activity at saturating (**a**) ATP and (**b**) flavin mononucleotide (FMN)) concentrations, and for the FADpp activity at saturating (**c**) PPi and (**d**) flavin adenine dinucleotide (FAD) concentrations of WT (●), R161A (■), R161D (□) and T165A (Δ) *Ca*FADS variants.

Replacements at any of the two His residues and at N125 prevented transformation of FMN into FAD, indicating that they are crucial for FMNAT catalytic activity. Substitutions at R161 allowed FAD production but a decrease in catalytic efficiency was observed, especially for R161D (Table 1, Figure 2a,b). Mutations in the consensus xSSTxxR motif prevented transformation of FMN when Asp was substituted for S164 or T165, while deleterious effects on the catalytic efficiency were observed for the Ala variants (Table 1, Figure 2a,b). Lack of activity exhibited by some variants might be due to either low *k*_cat_ or high *K*_m_ values, but the binding studies reported below (Sections 2.6 and 2.7) confirm that the mutations particularly affect the *k*_cat_ parameter.

### 2.4. Effects of Mutations on the FADpp Activity of CaFADS

The values of *k*_cat_, *K*_m_^FAD^, and *K*_m_^PPi^ of WT *Ca*FADS for the transformation of FAD and PPi into FMN and ATP are 13 min^−1^, 1.4 μM, and 114 μM respectively (Table 2, Figure 2c,d), in agreement with data previously reported [32]. Catalytic efficiencies were in a similar range to those for the forward reaction. No reaction was detected in the absence of PPi, indicating that *Ca*FADS has no FAD hydrolase activity. The mutations produced deleterious effects on FADpp activity, preventing in most cases product formation. Activity was only detected for N125D, R161A, R161D, S164A and T165A but, in general, the catalytic efficiency decreased significantly as a consequence of a reduction in *k*_cat_ and an increase in *K*_m_^PPi^ (Table 2, Figure 2c,d).

### 2.5. Effects of Mutations on the RFK Activity of CaFADS

The RFK activity of WT *Ca*FADS exhibits an inhibition profile on the RF substrate at saturated ATP concentrations that is similarly observed in all the mutants here produced (not shown) [9,20]. A fit of the data to an equation describing the dead-end inhibition by excess of RF, generated the inhibition constant (*K*_i_), *K*_m_^RF^, *k*_cat_, and catalytic efficiency values for each variant within a factor of two of those of the WT enzyme (Table S2). Similar profiles and kinetic parameters for RFK activity of WT *Ca*FADS were also observed for all the mutants when assayed at different ATP concentrations while keeping the RF one fixed. Therefore, mutations at H28, H31, N125, R161, S164 and T165 at the FMNAT-module of *Ca*FADS do not produce major deleterious effects on the RFK activity, but only slightly modulate binding and transformation of substrates. Among the residues here mutated only T165 is involved in the interaction with the RFK-module of a neighbouring protomer by establishing one H-bond with D298 (Figure S4). However, the region holding D298 is far from the RFK-module active site and does not involve any of the flexible regions of the RFK-module related with this activity.

### 2.6. Effects of Mutations on Binding Parameters of the Flavins to CaFADS

**Figure 3:**
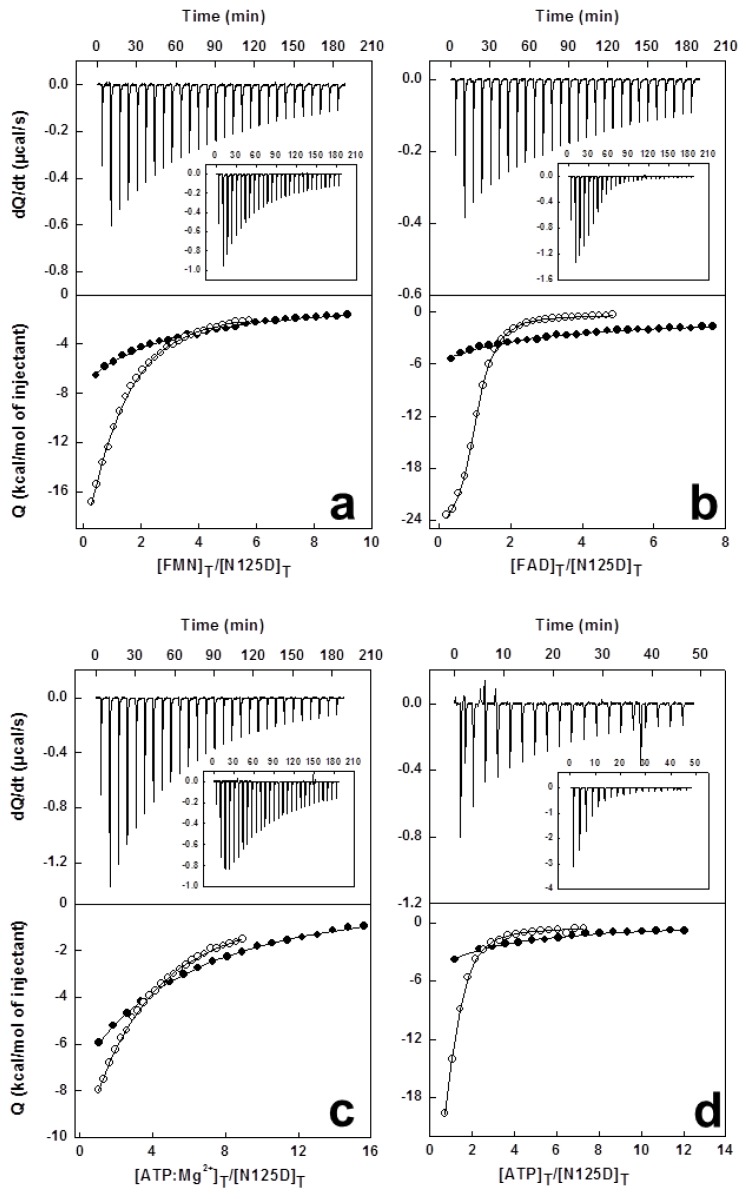
Calorimetric titrations of N125D and WT *Ca*FADS (~20 μM) with: (**a**) FMN (200 μM), (**b**) FAD (200 μM), and (**c**,**d**) ATP (300 μM). Upper panels show thermograms for the N125D and WT (inset) interaction, and lower panels the corresponding binding isotherms with integrated heats for N125D (●) and WT (○). Experiments carried out in 20 mM PIPES, 10 mM MgCl_2_, pH 7.0 for (**a**–**c**) and in absence of MgCl_2_ for (**d**).

**Figure 4:**
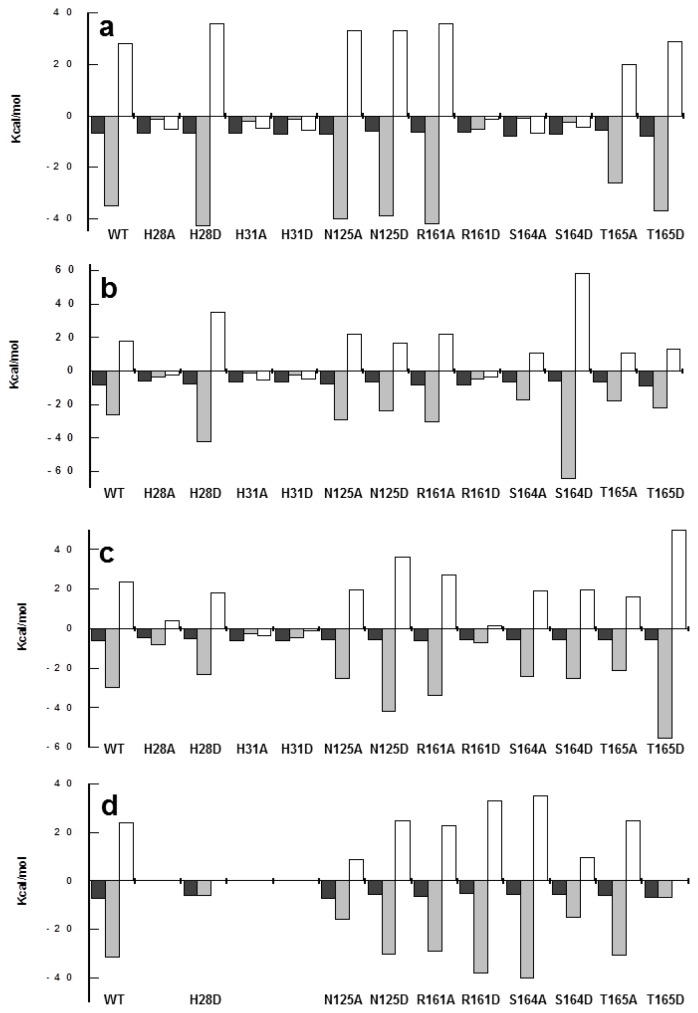
Thermodynamic dissection of the interaction of the *Ca*FADS forms with (**a**) FMN, (**b**) FAD and (**c**,**d**) ATP. The binding Gibbs energy (Δ*G*), enthalpy (Δ*H*), and entropy (−TΔ*S*) are represented by dark grey, light grey and white bars, respectively. Conditions are: 20 mM PIPES, 10 mM MgCl_2_, pH 7.0 for (**a**–**c**) and in absence of MgCl_2_ for (**d**).

ITC analysis of the interaction of WT *Ca*FADS with FMN and FAD was consistent with a single binding site at the FMNAT-module for both ligands, with dissociation constant (*K*_d_) values of 7.8 and 0.7 μM, respectively (Table 3) [9,20,21]. All mutants maintained this single flavin binding site, but binding enthalpies for the interaction of FMN and FAD to H28A, H31A and H31D *Ca*FADS and of FMN to R161D, S164A and S164D *Ca*FADS are significantly reduced (Table S3, Figures 3a,b, 4a,b and S5).

Substitution of histidines in the consensus motif 28-HxGH-31 had minor effects on the *Ca*FADS affinity for FMN. Larger effects were observed on the FAD affinity, with *K*_d_^FAD^ values up to 65-fold higher than for WT (Table 3). Binding of FMN and FAD to N125D *Ca*FADS was 5- and 40-fold weaker, respectively, with respect to WT (Table 3, Figure 3a,b). Noticeably, when Asp substituted for R161 only a small fraction of the enzyme molecules appeared able to bind both flavins, while replacement with Ala did not significantly affect this parameter. Mutations at R161 produced a 2.5-fold decrease in the affinity for FMN (Table 3). Substitution of S164 resulted in *K*_d_^FMN^ values only slightly smaller than for WT *Ca*FADS, but the affinity for FAD decreased by 30-fold (Table 3). Finally, replacement of T165 with Ala decreased the *Ca*FADS affinity for FMN and FAD by ~15-fold, while slightly increasing upon replacement with Asp (Table 3).

Binding of both flavins to WT *Ca*FADS was driven by a large enthalpic change with a high cost in entropy (Figure 4a,b, Table S3). Three of the four histidine replacements at 28-HxGH-31 altered the profile for FMN binding, now driven by a very low enthalpic contribution and a favourable entropic driving force. The same behavior was observed for R161D and mutants at S164 (Figure 4a). The thermodynamic profile for the interaction of FAD was similar for all the variants. The only exceptions were H28A, H31A and H31D, which showed a considerably decreased enthalpic contribution while the entropy became favourable (Figure 4b). In addition, changes in the magnitude of both contributions are observed for variants R161D, and, especially, S164D (Figure 4b). It is remarkable that in S164D, the effect on the thermodynamic parameters was opposite for the interaction with either FMN or FAD, suggesting that in this variant, the overall mode of interaction and the residues involved in their binding might differ. Particularly, the small values of the enthalpic contributions to binding suggest that the mutations prevent the formation of electrostatic interactions and H-bonds.

### 2.7. Effects of Mutations on the ATP Binding Parameters to CaFADS

Two independent ATP binding sites were detected for WT *Ca*FADS in the presence of 10 mM Mg^2+^ with an average *K*_d_^ATP^ value (*K*_d,av_^ATP:Mg^) of 30 μM (Table 3) [9,20,21]. In the absence of magnesium, only one binding site, identified in the FMNAT-module, was detected with a *K*_d_^ATP^ of 5.5 μM (Table 3) [9,20]. Two ATP:Mg^2+^ binding sites were also detected for all the *Ca*FADS variants, but in general mutations produced deleterious effects in the affinity. Only H31A and R161A showed *K*_d,av_^ATP^ values in the WT range. In general, *K*_d,av_^ATP^ were more than 3-fold higher than for WT, and up to 20-fold in H28A (Table 3, Figure 3c).

In the absence of magnesium the interaction of variants at H28 and H31, if any, is particularly weak. Only H28D showed appreciable binding, although the affinity considerably decreased with respect to WT (*K*_d_^ATP^ ~7-fold higher). The rest of the variants kept the ability to bind one ATP molecule at the FMNAT site, but with the only exception of the N125A and T165D mutants, the affinity for the adenine nucleotide was particularly weak (close to 20-fold for N125D, R161D, S164A and S164D) (Table 3, Figure 3d). These observations, together with the considerable decrease in the magnitude of the enthalpic and entropic contributions for ATP binding to the variants in the HxGH motif (Figure 4c), can be explained by the ATP binding site in the FMNAT-module being significantly altered by the mutations. Moreover, no good correlation was observed between *K*_d_^ATP^ and *K*_m_^ATP^ for active variants (with *K*_m_^ATP^ values lower than *K*_d_^ATP^ values (Tables 1 and 3)), suggesting that in the *Ca*FADS mutants, reactions might proceed through a different mechanism from WT. Once again these results indicate the importance of polarity in the cavity of the catalytic site for both binding of ligands and catalysis.

### 2.8. Structural Bases for the Role of Key Residues for the FMNAT and FADpp Activities of CaFADS

**Figure 5:**
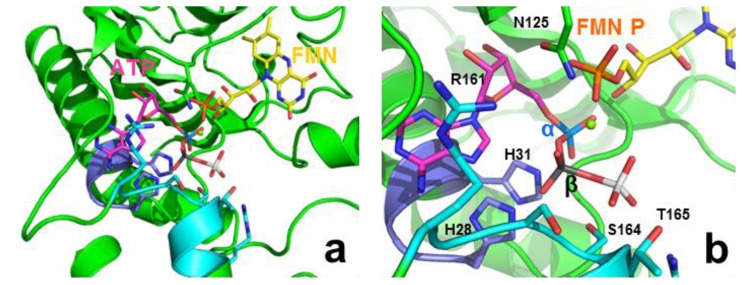
(**a**) Putative positioning of ATP, Mg^2+^ and FMN substrates in the FMNAT-module of *Ca*FADS. Structure produced by comparing the *Ca*FADS crystal structure with those of the NMNAT from *M. jannaschii* (PDB code 1F9A) and *M. thermoautotropicum* (PDB code 1EJ2) in complexes with Mg^2+^: ATP and NAD^+^, respectively, and the FMNAT-module of *Thermotoga maritima* FADS in complex with AMP (PDB code 1T6Y). FMN and ATP are shown by sticks with carbons in yellow and magenta, respectively, while Mg^2+^ is shown as a green sphere. The phosphate of FMN is shown in orange, while those of ATP are in blue (α), grey (β) and white (γ). (**b**) Model showing a putative organisation of the different phosphate groups at the catalytic site.

Catalysis by enzymes of the α/β phosphodiesterase NT family is proposed to achieve nucleotidylation of substrates without the direct involvement of active site residues in covalent or acid-base catalysis [27,30,33,34]. Therefore, in FADS the adenylylation reaction is expected to occur via a nucleophilic attack by the 5′-P of the FMN on the α-P of ATP, releasing FAD and PPi [31,35]. Superposition of the FMNAT-module of *Ca*FADS with some NTs in complex with substrates allowed tentative positioning of ATP and FMN (Figure 5a) [18].

The model suggests that H28 and H31 might contribute to allocate the α-P of ATP in the catalytically competent complex (Figure 5b), in agreement with the lack of FMNAT and FADpp activities, when both histidines, particularly H31, are replaced (Tables 1 and 2). Binding characterisation additionally supports this model, since replacement of both histidines alters the enthalpic/entropic ATP binding profiles (Figure 4c,d). The isoalloxazine and ribityl of FMN or FAD are proposed to bind in a cavity formed by several flexible protein loops (Figures 1a and 5a) [18]. *In silico* models suggest that H28, H31, N125 and R161 might orientate the phosphate of FMN towards the α-P of ATP during the FMNAT activity (Figure 5b), and the PPi portion of FAD for the FADpp one. Removal of H31 will prevent a favourable interaction, which apparently is critical to allocate the phosphate of FMN at the catalytic site. This is further supported by the, in general, low FMN and FAD binding enthalpy in these mutants (Figure 4a,b) while difference spectra confirm that the isoalloxazine portion is still able to interact with the FMNAT-module (Figure S3). Therefore, in WT *Ca*FADS, the enthalpic contribution to FMN binding is mainly provided by interaction of the HxGH motif, particularly H31, with the phosphates. This is in agreement with the fact that removal of H31 has a similar deleterious effect on the enthalpic contribution as removal of the phosphate of FMN for RF binding [21]. On the other hand, the entropy changes additionally suggest a more dynamic interaction, in agreement with conformational changes in the environment of some aromatic side chains at the FMNAT-module modifying the binding organisation of the substrates (Figure S2). Modeling of the position of ATP suggests that its adenine ring would be inserted at the bottom of the cavity establishing H-bonds with main chain atoms of some hydrophobic residues [18], while the oxygen connecting the α-P and β-P of ATP might be situated at the interacting distance of the Nɛ of H31 (Figure 5b).

Mutational studies on the equivalent histidines in CTP-glycerol-3-phosphate and CTP-phosphocholine synthase from *Bacillus subtillis*, as well as in human and murine 3′-phosphoadenosine 5′-phosphosulfate synthases, reported deleterious effects on *k*_cat_, with minor changes in *K*_m_[22,23]. Alanine substitutions in *Methanobacterium thermoautotropicum* nicotinamide mononucleotide adenylyltransferase (NMNAT) produced variants with some activity, and the comparative analysis suggested that the second histidine was more relevant as the catalytic group [26,28,29]. Our data indicate that, in *Ca*FADS, this motif is critical both for the catalytically competent ternary complex to be formed and for the catalysis itself, suggesting both histidines might be involved in the stabilisation of the pentacoordinate transition state arising from nucleophilic attack at the α-P of ATP [22,34]. The lack of activity made it impossible to determine, from the kinetic parameters, which of them is more relevant. However, binding parameters and the structural model suggest a more important contribution for H31, while H28 would stabilise the position of H31 (through stacking and H-bond interactions, as reported for other proteins of the NT superfamily) [26,29].

The model for the complex of the FMNAT-module with ATP and FMN, would putatively locate N125 at an adequate distance to interact both with the α-P of ATP and with the phosphate of FMN (equivalent position to the PPi in the crystallographic structure) through H-bonds (Figure 5b). Either replacement of N125 by Ala or Asp produces variants unable to transform FMN into FAD and, with a very low efficiency in the FADpp activity (Tables 1 and 2). The interaction of N125D with flavins and ATP is clearly altered (Table 3, Figure 3). By contrast, although the presence of an Ala at this position still allows binding it cannot stabilise the phosphates, and substrates are not able to achieve a catalytically competent interaction. Since the FADS sequence alignments show that in general either Asn or Asp can be found in this position [17], the properties of this position might be key for each particular FADS species. Therefore, both our structural models and biochemical data suggest that N125 contributes in locating and orientating both ligands as a previous step for catalysis, and envisages a possible involvement in the stabilisation of the transition state.

With regard to the 161-xxSSTxxR-168 motif, replacements at S164 and T165 also produce important deleterious effects in the FMNAT and FADpp activities of *Ca*FADS (Tables 1 and 2). Mutations at S164, despite allowing FMN binding, appear to produce a different protein-ligand organisation in the cavity (Table 3, Figure 4). Moreover, interactions with ATP and, especially, with FAD were clearly altered, indicating that this serine must stabilise ligand binding, probably by H-bond contact. In fact, introduction of a negatively charge residue produces a variant completely inactive to both activities, but able to bind flavins and ATP (Tables 1–3). Such behavior might be explained by an electrostatic repulsion between the introduced aspartate and the phosphates that avoids their proper position in the active site. The low catalytic efficiencies observed when an Ala replaced S164 supports that this serine might provide polarity for transition state stabilisation. Replacement of T165 by an Ala produced deleterious effects in the interaction with FMN and FAD (Table 3). Despite our kinetic parameters indicating that T165 is critical for substrate binding and catalysis (Tables 1 and 2, Figure 2), the structural model does not envisage any apparent interaction with ligands. Therefore, the interaction of the FMNAT-module with its substrates might include conformational changes to allow T165 to play a role in the catalytically competent complex or, alternatively, T165 might be involved in a conformational change necessary for binding and catalysis. Finally, the position of R161 is usually occupied by a positively charged side chain, Arg or Lys, in the FADS family. However, non-polar residues, as Val and Ile, can be also found in FADSs and NTs [17]. Replacements at R161 in *Ca*FADS produce deleterious effects on *k*_cat_ (Tables 1 and 2, Figure 2), and only a few molecules of the R161D variant appear active to bind FAD, FMN and ATP. All together these observations might be explained by a folding difference inducing a significant energetic barrier for ligand binding that produces an effect unrelated to the enzyme catalytic mechanism itself. This is consistent with the absence of difference spectra for the interaction of this mutant with either FMN or FAD. These observations suggest a role for R161 in ATP binding and probably also in the stabilisation of the transient state during the reaction, as also proposed for the equivalent residue in NMNAT [28]. The structural model analysis does not envisage any direct interaction of this residue with ligands, suggesting conformational changes in the protein module upon ATP interaction.

## 3. Experimental Section

### 3.1. Biological Material

Mutations were introduced into the pET28a-*Ca*FADS plasmid [17] by using the QuikChange kit (Stratagene, La Jolla, CA, USA) in combination with adequate synthetic oligonucleotides (Supplementary Material), and verified by DNA sequence analysis. WT and mutated *Ca*FADS were over-expressed in *E. coli* and purified as described [17]. Samples were dialysed in 20 mM PIPES, 10 mM MgCl_2_, pH 7.0 and stored at −80 °C. A gel filtration chromatography with HiPrep 26/60 Sephacryl S-200 HR column (GE Healthcare, Uppsala, Sweden) was used to separate the monomeric fraction [18,20].

### 3.2. Spectral Analysis

CD spectra were recorded in a Chirascan spectropolarimeter (Applied Photophysics Ltd., Leatherhead, Surrey, UK) at 25 °C. 5 μM *Ca*FADS in 5 mM PIPES, 10 mM MgCl2, pH 7.0 and 20 μM *Ca*FADS in 20 mM PIPES, 10 mM MgCl_2_, pH 7.0 were used in the far-UV (cuvette path length, 0.1 cm) and near-UV CD (0.4 cm), respectively. Difference spectra (Figure S3) were obtained upon addition of saturated concentrations for each one of the different ligands (50–100 μM FAD or FMN and 20–40 μM RF) in 20 mM PIPES, 10 mM MgCl_2_, pH 7.0 to samples of either *Ca*FADS (3–4 μM) or the *Ca*FADS:ADP (3–4 μM with final ADP concentration of ~450 μM) complexes for the different variants.

### 3.3. Determination of Steady-State Kinetics Parameters for the RFK, FMNAT and FADpp Activities of FADS

The *Ca*FADS RFK activity was measured at a final volume of 500 μL in 20 mM PIPES, 0.8 mM MgCl_2_, pH 7.0 containing 0.5–45 μM RF, 10–400 μM ATP. The reaction mixture was pre-incubated at 37 °C and the reaction was initiated by addition of 20–50 nM of the enzyme. The mixture was incubated at 37 °C for periods ranging from 0.5–2 min and, finally, the reaction was stopped by heating at 100 °C for 5 min [20,21]. The protein was removed from the reaction mixture by centrifugation, and the supernatant composition was analysed using an Alliance HPLC system (Waters, Milford, MA, USA) equipped with a 2707 autosampler. An aliquot of 20 μL of the filtered solution was applied to a HSST3 column (4.6 × 150 mm, 3.5 μm, Waters), preceded by a pre-column (4.6 × 20 mm, 3.5 μm, Waters) of the same material. The chromatography was developed at 1 mL/min with a 6-min isocratic program of methanol 40% (v/v) in 5 mM ammonium acetate (pH 6.0). Detection of flavins was carried out using a 2475 Multi λ fluorescence detector (Waters, Milford, MA, USA), an excitation wavelength of 470 nm and emission of 530 nm. Under these conditions the retention times for the flavins were 2.25 min for FAD, 3.43 min for FMN, and 5.50 min for RF. The amounts of produced FMN or/and FAD were determined from their corresponding standard curves obtained under the same conditions. Kinetic data obtained for one substrate at saturated concentrations of the second one (nanomol of flavin transformed per minute) were interpreted using the Michaelis-Menten kinetic model, obtaining *k*_cat_ and *K*_m_ with errors of ±10%. A model describing the substrate inhibition effect produced in a bi-substrate enzyme kinetic was used to interpret the kinetic evolution of the RFK activity on RF concentration (see [20] and Supplementary Material) [36]. In these situations, errors in the apparent *K*_m_ and *k*_cat_ (^app^*K*_m_ and ^app^*k*_cat_) increased with *K*_i_ getting closer to *K*_m_^S^[20].

The *Ca*FADS FMNAT and FADpp activities were fluorimetrically measured using a continuous assay. Time dependent measurements were performed at a final volume of 1 mL in 20 mM PIPES, 10 mM MgCl_2_, pH 7.0, containing 3–8 μM FMN/FAD, 10–400 μM ATP/PPi and 20–40 nM of enzyme at 37 °C (higher concentrations for variants with low activity). Measurements were performed in a Cary Eclipse spectrophotofluorimeter with excitation and emission wavelengths at 420 nm and 530 nm, respectively. FAD and FMN fluorescence were individually calibrated using standard solutions. The rate of FAD formation was calculated from the rate of fluorescence decrease (Δ*F*/Δ*t*), measured as the tangent to the initial part of the experimental curve by applying the equation:

(1) $$\upsilon_{0}=\frac{\Delta F}{\Delta t\cdot (K_{\text{FMN}}-K_{\text{FAD}})}$$

where Δ*F* is the decrease in the value of the fluorescence expressed in arbitrary units, Δ*t* is the measurement of reaction time expressed in per minute, and *K*_FMN_ and *K*_FAD_ are the FMN and FAD fluorescent rate constants expressed in per micromolar [37]. The kinetic data obtained for one substrate at saturated concentrations of the second one were interpreted using the Michaelis-Menten kinetic model. Data are media of at least three different experiments allowing for estimation of *k*_cat_ and *K*_m_ errors of ±10%.

### 3.4. Differential Scanning Calorimetry (DSC)

Thermal stability was determined with a VP-DSC microcalorimeter (MicroCal LLC, Northampton, MA, USA). Protein samples and reference solutions were degassed and carefully loaded into the cells to avoid bubble formation. The baseline of the instrument was routinely recorded before the experiments with both cells filled with buffer. Thermal denaturation scans were performed with 20 μM FADS solutions in 20 mM PIPES, 10 mM MgCl_2_, pH 7.0, at a scanning rate of 1 °C/min from 10 °C to 80 °C. Errors in the *T*_m,app_ data are within ±0.3–0.5 °C.

### 3.5. High Sensitivity Isothermal Titration Calorimetry (ITC)

Measurements were carried out using a high precision VP-ITC (MicroCal LLC, Northampton, MA, USA) following the procedure described previously [20,21]. Typically, 200 μM FAD or FMN and 300 μM ATP solutions were used to titrate ~20 μM *Ca*FADS. Ligand and *Ca*FADS were dissolved in 20 mM PIPES, pH 7.0, at the indicated MgCl_2_ concentration. Control titrations were performed by injecting ligand into buffer solution. The association constant, the enthalpy change (Δ*H*) and the stoichiometry, or their average values, were obtained through non-linear regression of the experimental data to a home derived model for one or two independent binding sites implemented in Origin 7.0 (OriginLab Corporation, Northampton, MA, 2002) (see Supplementary Material). The *K*_d_, the free energy change (Δ*G*), and the entropy change (Δ*S*) were obtained from basic thermodynamic relationships. Experiments were performed in duplicate or triplicate. The errors considered in the measured parameters (±15% in *K*_d_ and ±0.3 kcal/mol in Δ*H* and −TΔ*S*) were taken as larger than the standard deviation between replicates and the numerical error after fitting analysis.

## 4. Conclusions

A cluster of positive residues has evolved to properly orientate the interacting substrates and to intervene directly in the stabilisation of the catalytic competent complexes for the FMNAT and FADpp reactions catalysed by the FMNAT-module of *Ca*FADS. Replacements, introducing negatively charged residues at the 28-HxGH-31, 123-Gx(D/N)-125 and 161-RxSSTxxR-168 motifs of *Ca*FADS produce, in general, deleterious effects either by preventing nucleophilic attack or by causing an incorrect accommodation of the substrates in the active site for catalysis. Therefore, these motifs play an essential role in the formation of the catalytically competent complex, as well as in the catalysis for the FMNAT and FADpp activities. Since these motifs are clearly critical for activity and have no sequence or structural similarity in eukaryotic FMNATs [18,38], the cavity they conform is a particularly attractive target for developing specific drug therapies through the design of structure-based inhibitors of the FAD production in microorganisms [39].

## Supplementary Information

### 1. Experimental Section

#### 1.1. Biological Material

Mutations were introduced into the pET28a-*Ca*FADS plasmid codifying for the WT *Ca*FADS [20] by using the QuikChange mutagenesis kit (Stratagene, La Jolla, CA, USA) in combination with the following synthetic oligonucleotides (modified bases shown in bold):

5′-GTGTCTTCGACGGCGTG**GCG**CGCGGGCATCAGAAATTG-3′ for H28A;
5′-GTGTCTTCGACGGCGTG**G**A**C**CGCGGGCATCAGAAATTG-3′ for H28D;
5′-GGCGTGCATCGCGGG**GCG**CAGAAATTGATTAATGCC-3′ for H31A;
5′-GGCGTGCATCGCGGG**G**A**C**CAGAAATTGATTAATGC-3′ for H31D;
5′-GACGATGAAGGCGTG**GC**GATCTCTTCCACGACC-3′ for R161A;
5′-CTTGACGATGAAGGCGTG**GAC**ATCTCTTCCACGACCG-3′ for R161D;
5′-GTGAGGATCTCT**G**C**G**ACGACCGTGCGCGAGTTTC-3′ for S164A;
5′-CGTGAGGATCTCT**GAT**ACGACCGTGCGCGAGTTTC-3′ for S164D;
5′-GTGAGGATCTCTTCC**G**C**C**ACCGTGCGCGAGTTTCTATC-3′ for T165A and;
5′-GTGAGGATCTCTTCC**GAT**ACCGTGCGCGAGTTTCTATC-3′ for T165D.

Plasmids pET28a-*Ca*FADS for N125A and N125D were obtained from Mutagenex^®^.

#### 1.2. Kinetic Analysis

When RF inhibition was detected for the RFK activity, the experimental kinetic profiles obtained when varying the flavin concentration at saturated ATP were fit to the equation describing the inhibition effect produced in a bi-substrate enzyme kinetics when two molecules of the substrate bind to the enzyme and one blocks the competent binding of the other, or when the product of the reaction is not released leading to a dead-end complex:

(S1) $$\frac{v}{{\left[\text{E}\right]}_{\text{T}}}=\frac{k_{\text{cat}}\;[\text{S}]}{K_{\text{m}}^{\text{s}}+[\text{S}](1+[\text{S}]/K_{\text{i}})}$$

In these cases, a decrease in velocity (reaction rate, ν, divided by the total concentration of enzyme, [E]_T_) is usually observed at concentrations of the varying substrate, [S], around or greater than the dissociation constant of the inhibitor, *K*_i_. Errors in the determined apparent *K*_m_ and *k*_cat_ (^app^*K*_m_ and ^app^*k_cat_*) will increase with *K*_i_ getting closer to *K*_m_^S^. Thus, for *K*_i_ values within a factor of two of *K*_m_^S^, errors in the determined parameters were ±35%, while they decreased to ±10% when *K*_i_ was more than 3-fold larger than *K*_m_^S^.

#### 1.3. Isothermal Titration Calorimetry

The association constant (*K*_a_), the enthalpy change (Δ*H*) and the stoichiometry (N), or their average values, were obtained through non-linear regression of the experimental data to a model for one or two independent binding sites implemented in Origin 7.0 (OriginLab Corporation, Northampton, MA, USA, 2002).

The concentrations of protein and ligand after injection *i* are given by:

(S2) $${\left[P\right]}_{T,i}=P_{0}\;{\left(1-\frac{v}{V_{0}}\right)}^{i}$$

(S3) $${\left[\text{L}\right]}_{T,i}=L_{0}\;{\left(1-\frac{v}{V_{0}}\right)}^{i}$$

where *P*_0_ and *L*_0_ are the initial concentration of protein in the cell and the concentration of ligand in the syringe, respectively, and *v* and *V*_0_ are the injection volume and the cell volume, respectively.

If the protein exhibits one ligand binding site (or two binding sites with similar thermodynamic binding parameters), the following equation must be solved for each injection *i*:

(S4) $${\left[L\right]}_{T,i}={\left[L\right]}_{i}+{\left[P\right]}_{T,i}\frac{K_{\text{a}}{\left[L\right]}_{i}}{1+K_{\text{a}}{\left[L\right]}_{i}}$$

which provides the concentration of free ligand after each injection, [*L*]*_i_*, assuming a given value of the association constant *K*_a_. The concentration of protein-ligand complex formed up to injection *i* is calculated as follows:

(S5) $${\left[PL\right]}_{i}={\left[P\right]}_{T,i}\frac{K_{\text{a}}{\left[L\right]}_{i}}{1+K_{\text{a}}{\left[L\right]}_{i}}$$

and the heat associated with each injection is given by:

(S6) $$q_{i}=V_{0}\;\left(\Delta H\;\left({\left[PL\right]}_{i}-{\left[PL\right]}_{i-1}\;\left(1-\frac{v}{V_{0}}\right)\right)\right)+q_{d}$$

where Δ*H* is the enthalpy of ligand binding.

If the protein exhibits two different, independent ligand binding sites, the following equation must be solved for each injection *i*:

(S7) $${\left[L\right]}_{T,i}={\left[L\right]}_{i}+{\left[P\right]}_{T,i}\frac{(K_{\text{a}1}+K_{\text{a}2}){\left[L\right]}_{i}+2K_{\text{a}1}K_{\text{a}2}{\left[L\right]}_{i}^{2}}{1+(K_{\text{a}1}+K_{\text{a}2}){\left[L\right]}_{i}+K_{\text{a}1}K_{\text{a}2}{\left[L\right]}_{i}^{2}}$$

which provides the concentration of free ligand after each injection, [*L*]*_i_*, assuming given values of the association constants *K*_a1_ and *K*_a2_. The concentrations of the two protein-ligand complexes formed up to injection i are calculated as follows:

(S8) $$\begin{array}{l} {\left[PL\right]}_{i}={\left[P\right]}_{T,i}\frac{K_{\text{a}1}{\left[L\right]}_{i}}{1+K_{\text{a}1}{\left[L\right]}_{i}}\\ {\left[LP\right]}_{i}={\left[P\right]}_{T,i}\frac{K_{\text{a}2}{\left[L\right]}_{i}}{1+K_{\text{a}2}{\left[L\right]}_{i}} \end{array}$$

where we have distinguished between the two protein complexes, *PL* and *LP*. The heat associated with each injection is given by:

(S9) $$q_{i}=V_{0}\left(\Delta H_{1}\left({\left[PL\right]}_{i}-{\left[PL\right]}_{i-1}\left(1-\frac{v}{V_{0}}\right)\right)+\Delta H_{2}\left({\left[LP\right]}_{i}-{\left[LP\right]}_{i-1}\left(1-\frac{v}{V_{0}}\right)\right)\right)+q_{d}$$

where Δ*H*_1_ and Δ*H*_2_ are the enthalpies of ligand binding to each binding site in the protein.

Control titrations were performed injecting ligand into buffer solution for assessing ligand self-association. Although these experiments are routinely used for estimating the dilution heats in a protein-ligand titration, they often fail to reproduce those dilution heats. In addition, in low-affinity titration experiments where complete saturation is not reached, subtraction of ligand dilution control heats is very often problematic. We included an adjustable parameter, qd, in the fitting function to account for the dilution heats in each experiment.

The nominal protein concentration was normalised by a factor *n* (*nP*_0_, instead of *P*_0_) accounting for either the stoichiometry or the fraction of active (binding-competent) protein.

**Table S1:** *T*_m,app_ for the different *Ca*FADS forms obtained by DSC in 20 mM PIPES, 10 mM MgCl_2_, pH 7.0. Estimated errors in *T*_m,app_ were considered within ±0.3–0.5 °C, this value taken being larger than the standard deviation between three independent experiments and the numerical error after fitting the analysis of each experiment.

	*T* _m,app_ (°C)
WT	42.5
H28A	48.9
H28D	41.5
H31A	45.9
N125A	40.2
N125D	43.5
R161A	49.2
R161D	43.9
S164A	38.9
S164D	49.7
T165A	40.1
T165D	44.9

**Table S2:** Steady-state kinetic parameters for the RFK activity (RF + ATP → FMN + ADP) of the different *Ca*FADS forms. Data obtained at 37 °C in 20 mM PIPES, 0.8 mM MgCl_2_, pH 7.0.

	*k* _cat_ ^b^, ^c^ (min ^−1^)	*K* _m_ ^RF^ b, c (μM)	*K _i_* ^b^, ^c^ (μM)	*k* _cat_ */K* _m_ ^RF^ b (min ^−1^ μM ^−1^)	*k* _cat_ ^c^, ^d^ (min ^−1^)	*K* _m_ ^ATP^ c, d (μM)	*k* _cat_ */K* _m_ ^ATP^ d (min ^−1^ μM ^−1^)
WT a	301	13	4.0	23	68	14	4.9
H28A	427	23	1.8	18	45	14	3.1
H28D	287	25	1.9	12	55	12	4.7
H31A	169	4.5	6.3	37	58	14	4.3
N125A	111	1.7	4.6	65	46	24	1.9
N125D	415	16	3.3	26	80	35	2.3
R161A	300	12	5.1	25	65	12	5.6
R161D	300	13	5.1	23	68	11	6.1
S164A	259	8.8	4.4	29	59	12	5.0
S164D	185	5.6	5.8	33	55	10	5.3
T165A	171	10	3.2	17	66	12	5.4
T165D	180	7.7	4.1	23	45	11	4.1

a Data from [24].

b Parameters determined at saturated ATP concentrations.

c Inhibition by substrate prevented the determination of true parameters and the values here reported correspond to apparent constants; ^app^*k*_cat_ and ^app^*K*_m_. Estimated errors in ^app^*k*_cat_ and ^app^*K*_m_ values increased up to ±35%.

d Parameters estimated using a RF concentration exhibiting ~80% maximal activity before the maximum experimentally detected. Errors in *k*_cat_ and *K*_m_ were considered within ±10%, being this value taken larger than the standard deviation between three independent experiments and the numerical error after fitting analysis of each experiment to the Michaelis-Menten equation.

**Table S3:** Thermodynamic parameters for the interaction of *Ca*FADS WT and mutants with flavins determined by ITC. Data obtained at 25 °C in 20 mM PIPES, pH 7.0, at the indicated MgCl_2_ concentration. Errors in Δ*G*, Δ*H* and −TΔ*S* where ±0.3 kcal/mol, in general larger than the standard deviation between replicates and the numerical error after fitting the analysis.

	FADS:FMN 10 mM Mg ^2+^			FADS:FAD 10 mM Mg ^2+^			FADS:ATP 10 mM Mg ^2+^			FADS:ATP 0 mM Mg ^2+^		
				
	Δ *G* (kcal/mol)	Δ *H* (kcal/mol)	−TΔ *S* (kcal/mol)	Δ *G* (kcal/mol)	Δ *H* (kcal/mol)	−TΔ *S* (kcal/mol)	Δ *G* (kcal/mol)	Δ *H* (kcal/mol)	−TΔ *S* (kcal/mol)	Δ *G* (kcal/mol)	Δ *H* (kcal/mol)	−TΔ *S* (kcal/mol)
WT	−7.0	−35	28	−8.4	−26	18	−6.2	−30	23	−7.2	−31	24
H28A	−6.7	−1.4	−5.3	−5.9	−3.5	−2.4	−4.3	−8.3	4.0	---	---	---
H28D	−7.0	−43	36	−7.8	−42	35	−5.3	−23	18	−6.0	−6.1	0.1
H31A	−6.7	−2.0	−4.7	−6.5	−1.2	−5.3	−5.9	−2.6	−3.3	---	---	---
H31D	−7.1	−1.4	−5.7	−6.7	−2.1	−4.6	−5.9	−4.6	−1.3	---	---	---
N125A	−7.2	−40	33	−7.6	−29	22	−5.3	−25	20	−7.1	−16	8.7
N125D	−6.0	−39	33	−6.7	−24	17	−5.7	−42	36	−5.5	−30	25
R161A	−6.5	−42	36	−8.4	−30	22	−6.2	−34	27	−6.2	−29	23
R161D	−6.6	−5.2	−1.4	−8.3	−4.9	−3.4	−5.7	−7.2	1.5	−5.2	−38	33
S164A	−8.0	−1.1	−6.9	−6.3	−17	11	−5.4	−24	19	−5.5	−40	35
S164D	−7.2	−2.6	−4.6	−6.2	−64	58	−5.4	−25	20	−5.4	−15	9.6
T165A	−5.7	−26	20	−6.7	−18	11	−5.4	−21	16	−6.1	−31	25
T165D	−7.8	−37	29	−8.8	−22	13	−5.4	−55	50	−6.7	−6.9	0.2

Figure S1 (**a**) Scheme for the reactions catalysed by the RFK and the FMNAT modules of *Ca*FADS. (**b**) Cartoon representation and topology of the crystal structure of *Ca*FADS (2X0K). The *N*-terminal FMNAT and *C*-terminal RFK modules are coloured in green and orange, respectively. (**c**) Logo of sequence homology of the motifs putatively involved in the FMNAT catalytic activity in the FADS family. The sequence logo was produced using the server (available online: http://weblogo.berkeley.edu; accessed on15 February 2007).

Figure S2 Circular dichroism spectra (molar ellipticity per residue) in the (**a**) far-UV (5 μM *Ca*FADS in 5 mM PIPES, 10 mM MgCl_2_, pH 7.0 in a 0.1 cm path length cuvette) and (**b**) near-UV (20 μM *Ca*FADS in 20 mM PIPES, 10 mM MgCl_2_, pH 7.0 in a 0.4 cm path length cuvette) regions for WT (black), H28A (pink), H28D (red), H31A (green), H31D (grey), N125A (blue), N125D (cyan), R161A (magenta), R161D (yellow), S164A (purple), S164D (orange), T165A (dark green) and T165D (violet).

Figure S3 Visible difference spectra elicited upon addition to the *Ca*FADS variants (left) and their preformed *Ca*FADS:ADP complexes (right) of saturating concentrations of (**a** and **b**) RF, (**c** and **d**) FAD and (**e** and **f**) FMN for WT (black), H28D (red), H31A (green), N125A (blue), N125D (cyan), R161A (magenta), R161D (yellow), S164A (purple), S164D (orange), T165A (dark green) and T165D (violet).

Figure S4 (**a**) Surface representation of the hexamer of *Ca*FADS and detail of the RFK and FMNAT cavities. The docked ligands are represented by sticks. (**b**) Detail of the contacts between RFK (in cartoon and orange) and the FMNAT (in surface and green) between two protomers in the trimeric structure of *Ca*FADS.

Figure S5 Calorimetric titration of H28D and T165A *Ca*FADS (~20 μM) with (**a**–**e**) FMN (200 μM), (**b**–**f**) FAD (200 μM), (**c**–**g**) ATP (5 mM) and (**d**–**h**) ATP (200 μM) in absence of MgCl_2_. Upper panels show thermograms for the interaction and lower panels show the corresponding binding isotherms with integrated heats. Except for (d and h) the experiments were carried out in 20 mM PIPES, 10 mM MgCl_2_.

## Figures and Tables

**Table 1:** Steady-state kinetic parameters for the ATP:FMN adenylyltransferase (FMNAT) activity (FMN + ATP → FAD + PPi) of the different *Ca*FADS forms. Data obtained at 37 °C in 20 mM PIPES, 10 mM MgCl_2_, pH 7.0. Estimated errors in *k*_cat_ and *K*_m_ are considered within ±10%, this value taken being larger than the standard deviation between at least three independent experiments and the numerical error after fitting the analysis of each experiment to the Michaelis-Menten equation.

	*k*_cat_ (min^−1^)	*K*_m_^FMN^ (μM)	*K*_m_^ATP^ (μM)	*k*_cat_*/K*_m_^FMN^ (min^−1^ μM^−1^)	*k*_cat_*/K*_m_^ATP^ (min^−1^ μM^−1^)
WT a	17	1.2	36	14	0.48
H28A	<0.1	-	-	-	-
H28D	<0.1	-	-	-	-
H31A	<0.1	-	-	-	-
H31D	<0.1	-	-	-	-
N125A	<0.1	-	-	-	-
N125D	<0.1	-	-	-	-
R161A	15	6.1	1.0	2.4	15
R161D	3.9	12.0	8.3	0.32	0.47
S164A	17	20	86	0.84	0.20
S164D	<0.1	-	-	-	-
T165A	8.1	2.1	2.3	3.8	3.4
T165D	<0.1	-	-	-	-

a Data from [20].

**Table 2:** Steady-state kinetic parameters for the FADpp activity (FAD + PPi → FMN + ATP) of the *Ca*FADS forms. Data obtained at 37 °C in 20 mM PIPES, 10 mM MgCl_2_, pH 7.0. Errors in *k*_cat_ and *K*_m_ are estimated within ±10%, this value taken being larger than the standard deviation between three independent experiments and the numerical error after fitting the analysis of each experiment to the Michaelis-Menten equation.

	*k*_cat_ (min^−1^)	*K*_m_^FAD^ (μM)	*K*_m_^PPi^ (μM)	*k*_cat_*/K*_m_^FAD^ (min^−1^ μM^−1^)	*k*_cat_*/K*_m_^PPi^ (min^−1^ μM^−1^)
WT	13	1.4	114	8.6	0.110
H28A	<0.1	-	-	-	-
H28D	<0.1	-	-	-	-
H31A	<0.1	-	-	-	-
H31D	<0.1	-	-	-	-
N125A	<0.5	-	-	-	-
N125D	2.1	15	320	0.14	0.007
R161A	6.9	1.7	215	4.4	0.032
R161D	2.1	17	483	0.12	0.004
S164A	1.7	17	229	0.10	0.007
S164D	<0.1	-	-	-	-
T165A	1.7	1.3	537	1.2	0.003
T165D	<0.1	-	-	-	-

**Table 3:** Dissociation constants for the interaction of WT and mutated forms of *Ca*FADS with FMN, FAD and ATP as determined by ITC. Data obtained at 25 °C in 20 mM PIPES pH 7.0, at the indicated MgCl_2_ concentration. Errors in *K*_d_ are considered within ±15%, the value taken being larger than the standard deviation between at least three independent experiments and the numerical error after fitting analysis to the equation describing a model for either one or two independent binding sites.

	*K*_d_ (μM)			

	FADS:FMN 10 mM Mg^2+^	FADS:FAD 10 mM Mg^2+^	FADS:ATP 10 mM Mg^2+^	FADS:ATP 0 mM Mg^2+^
WT	7.8	0.7	30 a	5.5
H28A	13	48	>650 a	very weak
H28D	7.9	1.8	130 a	40
H31A	18	18	43 a	very weak
H31D	6.1	12	>50 a	very weak
N125A	5.6	2.7	123 a	6.4
N125D	38	30	70 a	91
R161A	18	0.8	27 a	27
R161D	14	1.0	61 a	164
S164A	1.4	26	111 a	86
S164D	5.3	27	104 a	114
T165A	65	11	118 a	32
T165D	2.0	0.4	103 a	11

a These parameters correspond to average dissociation constants (*K*_d,av_^ATP:Mg^) for two ATP:Mg^2+^ binding sites that cannot be independently determined [20].
