# Supplementary figures and images for: Role of Key Residues at the Flavin Mononucleotide (FMN):Adenylyltransferase Catalytic Site of the Bifunctional Riboflavin Kinase/Flavin Adenine Dinucleotide (FAD) Synthetase from Corynebacterium ammoniagenes
Source: Int J Mol Sci. 2012 Nov 8;13(11):14492–517. doi: 10.3390/ijms131114492 (PMC3509593; doi:10.3390/ijms131114492)

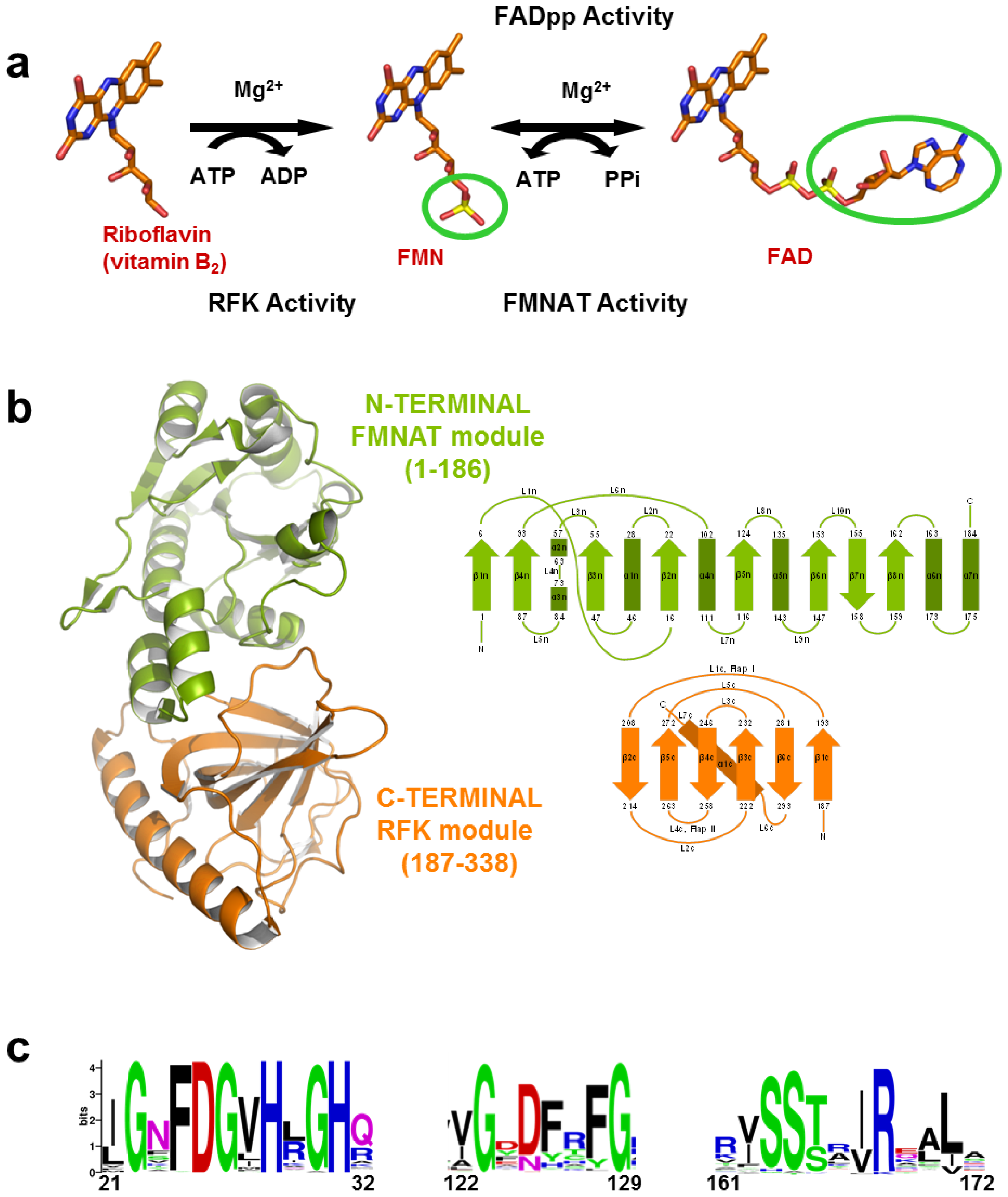

Supplement: Figure S1 — (a) Scheme for the reactions catalysed by the RFK and the FMNAT modules of CaFADS. (b) Cartoon representation and topology of the crystal structure of CaFADS (2X0K). The N-terminal FMNAT and C-terminal RFK modules are coloured in green and orange, respectively. (c) Logo of sequence homology of the motifs putatively involved in the FMNAT catalytic activity in the FADS family. The sequence logo was produced using the server (available online: http://weblogo.berkeley.edu; accessed on15 February 2007). [file ijms-13-14492s1.tif]

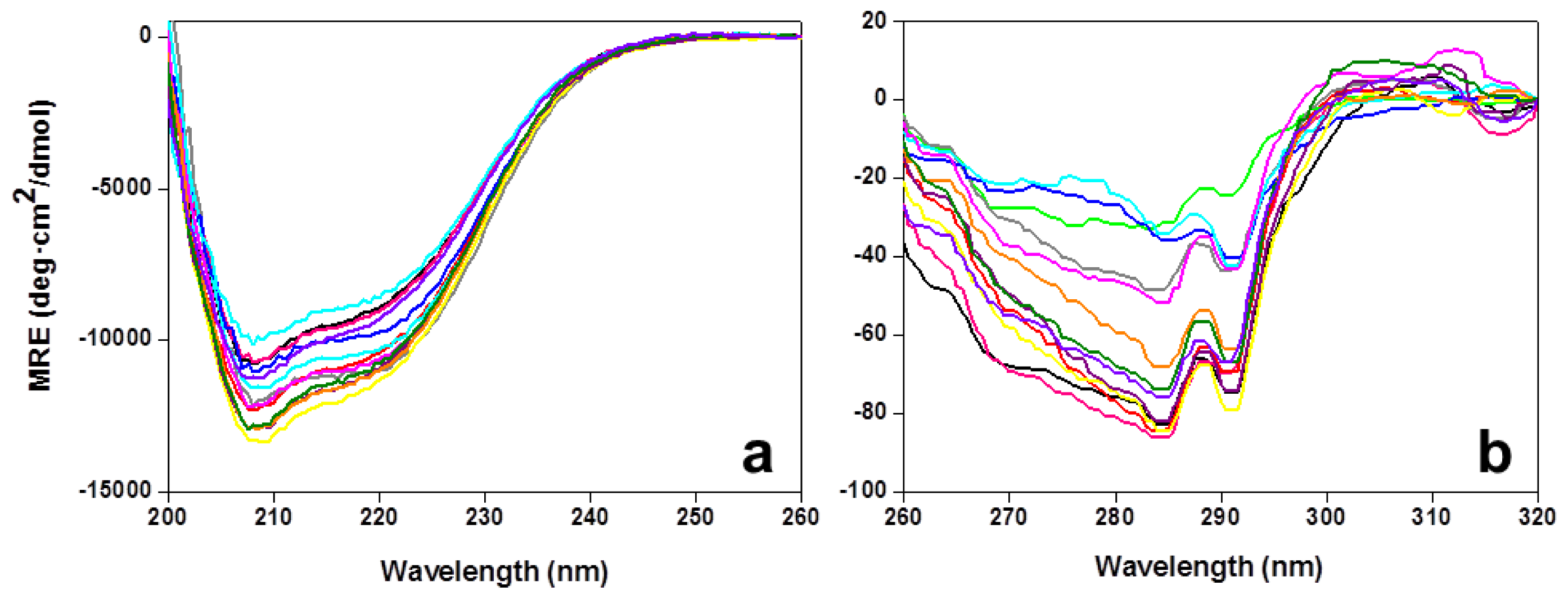

Supplement: Figure S2 — Circular dichroism spectra (molar ellipticity per residue) in the (a) far-UV (5 μM CaFADS in 5 mM PIPES, 10 mM MgCl2, pH 7.0 in a 0.1 cm path length cuvette) and (b) near-UV (20 μM CaFADS in 20 mM PIPES, 10 mM MgCl2, pH 7.0 in a 0.4 cm path length cuvette) regions for WT (black), H28A (pink), H28D (red), H31A (green), H31D (grey), N125A (blue), N125D (cyan), R161A (magenta), R161D (yellow), S164A (purple), S164D (orange), T165A (dark green) and T165D (violet). [file ijms-13-14492s2.tif]

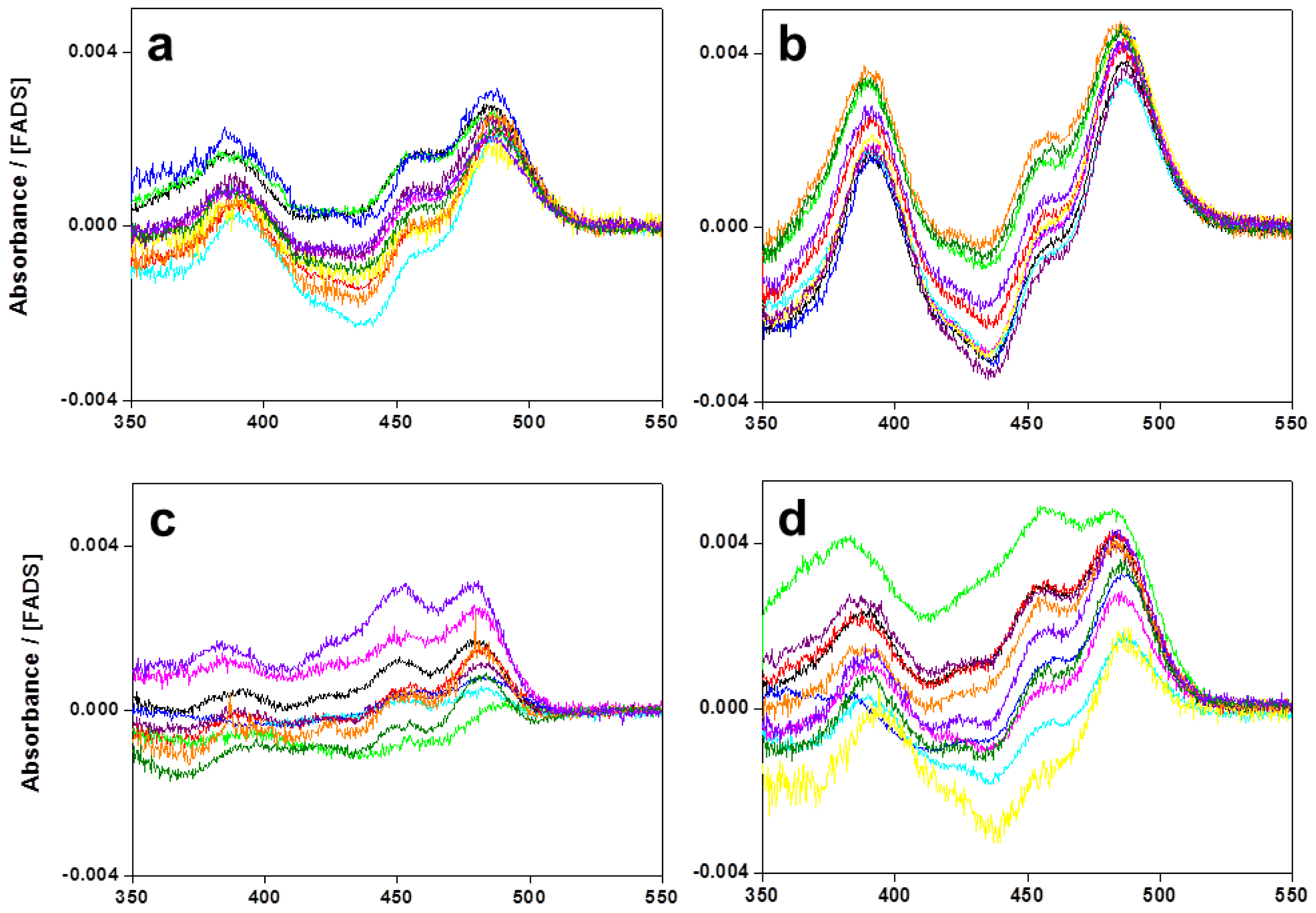

Supplement: Figure S3 — Visible difference spectra elicited upon addition to the CaFADS variants (left) and their preformed CaFADS:ADP complexes (right) of saturating concentrations of (a and b) RF, (c and d) FAD and (e and f) FMN for WT (black), H28D (red), H31A (green), N125A (blue), N125D (cyan), R161A (magenta), R161D (yellow), S164A (purple), S164D (orange), T165A (dark green) and T165D (violet). [file ijms-13-14492s3.tif]

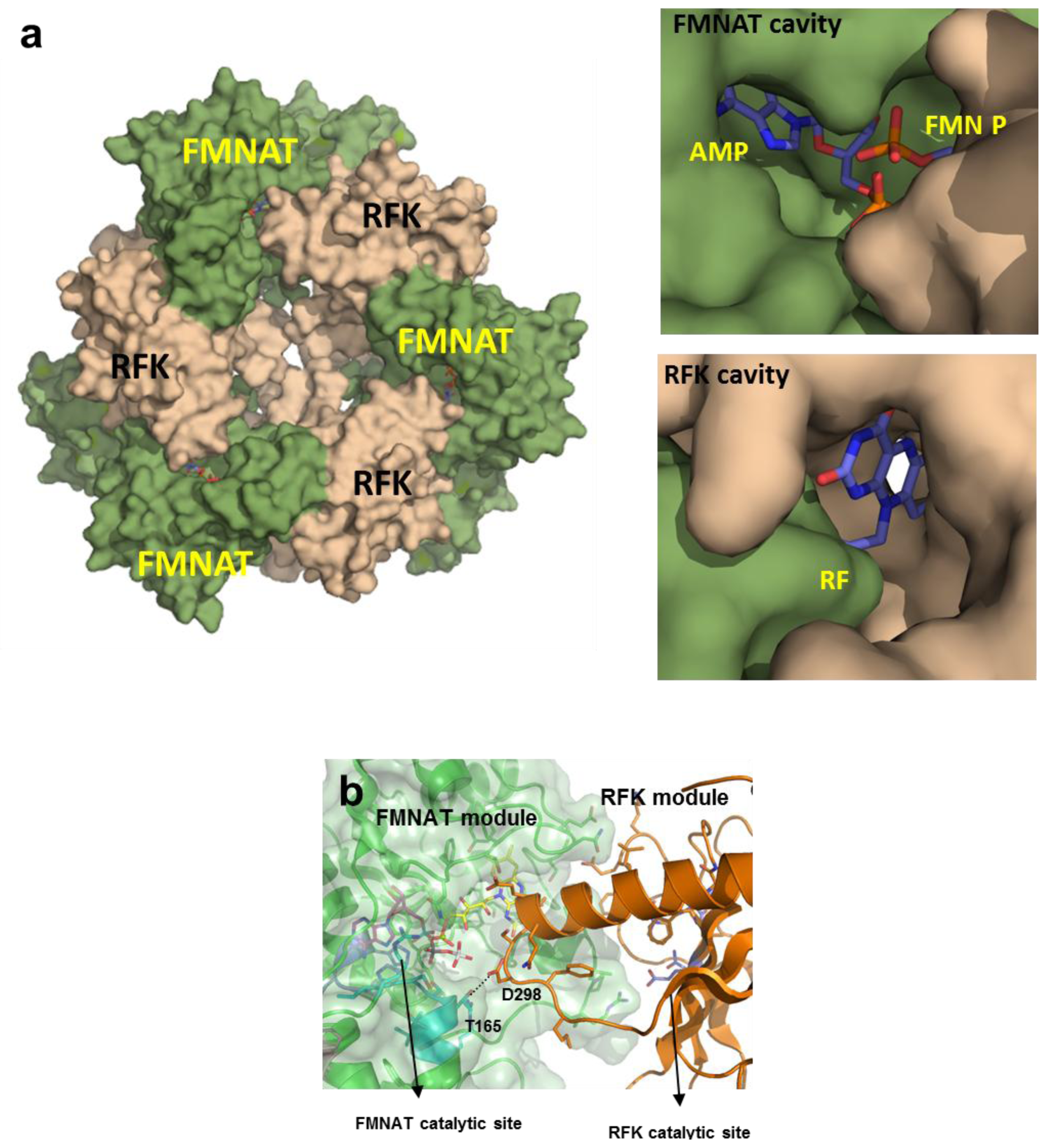

Supplement: Figure S4 — (a) Surface representation of the hexamer of CaFADS and detail of the RFK and FMNAT cavities. The docked ligands are represented by sticks. (b) Detail of the contacts between RFK (in cartoon and orange) and the FMNAT (in surface and green) between two protomers in the trimeric structure of CaFADS. [file ijms-13-14492s4.tif]

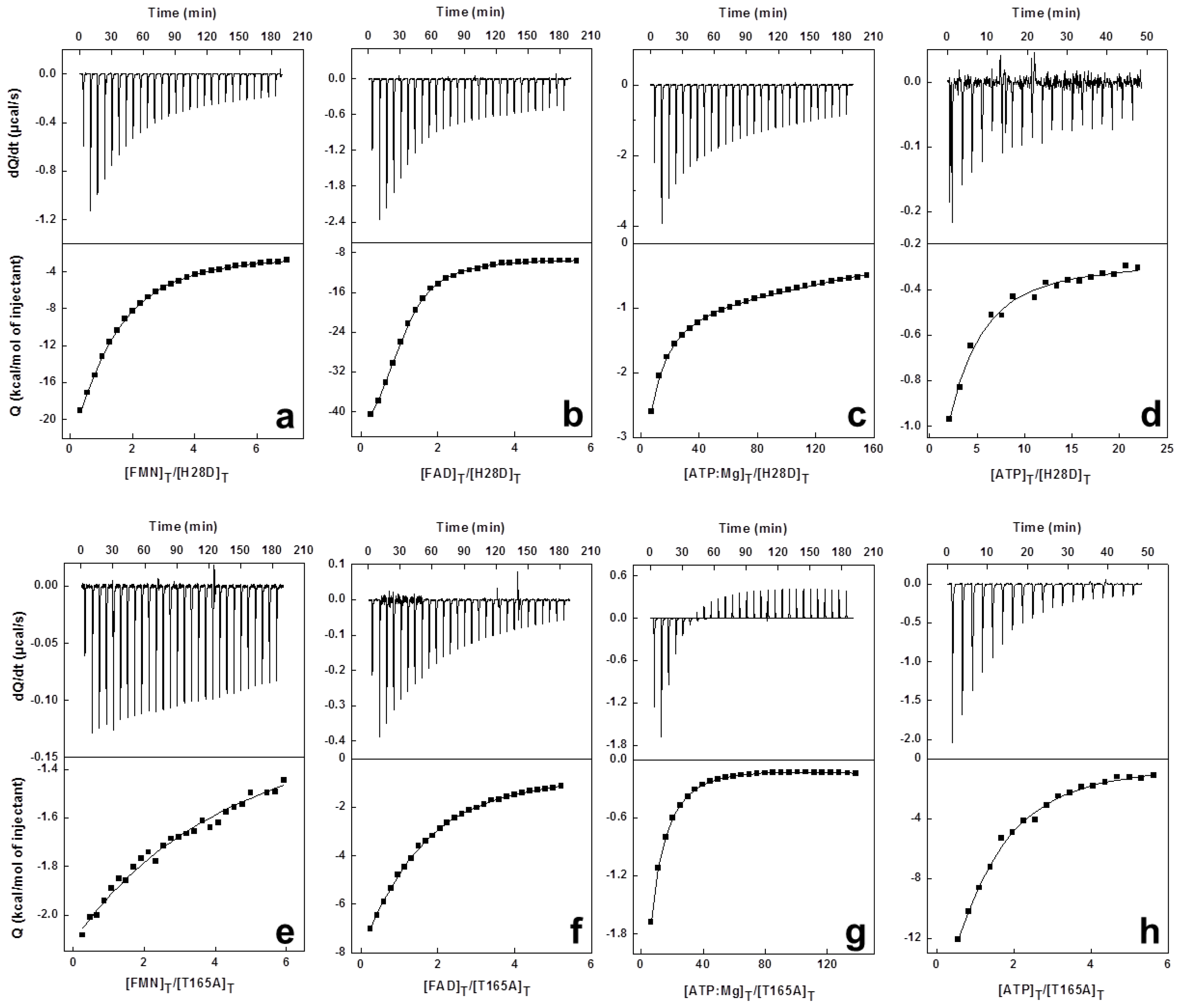

Supplement: Figure S5 — Calorimetric titration of H28D and T165A CaFADS (~20 μM) with (a–e) FMN (200 μM), (b–f) FAD (200 μM), (c–g) ATP (5 mM) and (d–h) ATP (200 μM) in absence of MgCl2. Upper panels show thermograms for the interaction and lower panels show the corresponding binding isotherms with integrated heats. Except for (d and h) the experiments were carried out in 20 mM PIPES, 10 mM MgCl2. [file ijms-13-14492s5.tif]
